# Molecular Screening of Staphylococcal Enterotoxin B Gene in Clinical Isolates

**Published:** 2011-09-23

**Authors:** Ramezan Ali Ataee, Ali Karami, Morteza Izadi, Aref Aghania, Mohammad Hossein Ataee

**Affiliations:** 1:Department of Medical Microbiology, Therapeutic Microbial Toxin Research Center, Faculty of Medicine, Baqiyatallah University of Medical Sciences, Tehran, Iran; 2: Molecular Biology Research Center, Baqiyatallah University of Medical Sciences, Tehran, Iran; 3: Department of Infectious Diseases, Health Research Center and, Faculty of Medicine, Baqiyatallah University of Medical Sciences, Tehran, Iran

**Keywords:** Screening, *Staphylococcus aureus*, Enterotoxin B, PCR, Western-Blot

## Abstract

**Objective:** The role of staphylococcal enterotoxin B (SEB) in food poisoning is well known, however its role in other diseases remains to be explored. The aim of this study is the molecular screening and characterization of the SEB gene in clinically isolated strains. **Materials and Methods:** In this experimentally study, 300 *Staphylococcus aureus (S. aureus)* strains isolated from clinical samples were assayed. The isolated strains were confirmed by conventional bacteriological methods. Polymerase chain reaction (PCR) was used to determine the enterotoxin B *(ent B)* gene. Assessment of toxin production in all strains that contained the *ent B* gene was then performed. Finally, using specific antibody against SEB, a Western-blot was applied to confirm detection of enterotoxin B production. **Results:** Results indicated that only 5% of the 300 clinically isolated *S. aureus* contained the *ent B* gene. All strains which contained the *ent B* gene produced a proteinous enterotoxin B. The results of sequence determination of the PCR product were compared with the gene bank database and 98% similarity was achieved. The results of the Western-blot confirmed that enterotoxin B was produced in strains that contained the *ent B* gene. **Conclusion:** The results of this study indicate that 5% of clinically isolated *S. aureus* strains produce enterotoxin B. Considering that the enterotoxin B is an important superantigen, it is possible that a delay in diagnosis and lack of early proper treatment can cause an incidence of late complications, particularly in staphylococcal chronic infections. For this reason, it is suggested that in addition to detecting bacteria, an enterotoxin B detection test should be performed to control its toxigenicity.

## Introduction

 Characteristics of *Staphylococcus aureus (S.aureus)* enterotoxin B (SEB) are well known (1). SEB is the most studied member of the staphylococcal enterotoxins produced by *S.aureus* and is responsible for staphylococcal food-poisoning. However, scientific data reveals some variable information. For example, different molecular weights for SEB have been reported (23-29 kDa and 31.4 kDa) (2). All of these reported proteins are heat stable, resistant to proteolytic digestion, and their pH range for tolerance is 4 to 10 (3, 4). SEB is a pyrogenic agent able to incapacitate human beings for up to two weeks with as low as a 0.5 µg/kg dose. Itsinherent stability, high morbidity rate, high intoxication effect, and ease of environmental dissemination were considered as important factors for its toxicity (5, 6). SEB also induces potent cytotoxic activity by intraepithelial lymphocytes (7). SEB has immunomodulatory effects in allergic airway disease and it facilitates sensitization to ovalbumin and consecutive bronchial inflammation with features of allergic asthma (8). SEB exerts direct pro-inflammatory and other pathological effects on human nasal epithelial cells, with induction of chemokine and growth factor release, resulting in the migration and prolonged survival of granulocytes in vitro (9,10). However, SEB acts as a superantigen and exhibits a dramatic proinflammatory impact on epithelial cells, which can be inhibited by the addition of glucocorticoids (11). SEB plays an important role in the treatment of certain cancers and autoimmune diseases (12). It is used as target molecule to find affinity ligands from a solid phase combinatorial peptide library, and then use of such ligands to detect, remove, and purify it by affinity chromatography. The derived peptide ligands can help us understand the interactions of SEB as a superantigen with a major histocompatibility complex class II molecule and T cell receptor (13, 14). Since SEB is an important agent of human disease, access to the methods of controlling diseases caused by SEB are important. Considering the above mentioned reasons, it is necessary to know the frequency of SEB producing strains. Our knowledge about the characteristics of molecular composition, biological properties, and genetics bases is low. The standard strains of the SEB producer *S.aureus* (for example ATCC 14448) or even a slight amount of this toxin (500µg) are currently not available in Iran and buying them is impossible from international vendors. Therefore, in order to carry out diagnostic techniques, it is necessarry to screen SEB producer strains and determine whether or not native strains that contain the gene of this toxin could be identified and demonstrate their characteristics. This would set up our knowledge base and impart the necessary skills in this important research subject and cater to the community's needs. The precise role of staphylococcal enterotoxins that cause delayed symptoms in different parts of the body is unknown. Thus, identification of enterotoxin producing strains isolated from areas other than the gastrointestinal tract is essential. The aim of this research is the molecular screening of gene encoded SEB in domestic strains and the confirmation of gene expression and enterotoxin B production.

## Materials and Methods

### Collection of bacterial strains

 From August 2008 to July 2010 a total 300 strains of *S.aureus* were isolated from clinical samplesand identified by bacteriological methods (gram stain, coagulase, mannitol fermentation and DNase tests). The isolates were stored on suitable maintenance media in our laboratory. Nontoxigenic strains (ATCC 6538 and PTCC 1435) were used as negative controls. The total protein of coagulase positive strains were measured by the Bradford method.

### DNA extraction

 DNA was extracted by the modified salting out method (15) as below: 1 ml of the overnight bacterial culture was centrifuged at 3000 × g for 5 minutes at room temperature. The supernatant was then discarded. Then, 500 µl STE buffer (Tris-HCl 10mM, NaCl 10 mM, EDTA 1 mM, pH =7.5) was added to the sediment and suspended by pipetting. After 10 minutes, 200 µl 2% SDS followed by 300 µl of 3M Natrium acetate were added and the tube was gently inverted 10 times. The tube was centrifuged at 5000 × g for 5 minutes and the supernatant was transferred to a new microfuge tube, which was then placed on ice. Cold isopropanol (1 ml) was added and mixed with the contents of the tube by gentle inversion. The tube was then placed in a −20°C freezer for 1 hour or more afterwhich the tube was centrifuged at 15000 × g for 5 minutes at 4°C. The supernatant was removed and 70% ethanol was added to the pellet. After several minutes, the tube was centrifuged for 2 minutes and the ethanol removed. The DNA pellet was allowed to dry for 5-10 minutes. The damp pellet was dissolved in 100 µl of TE buffer (pH= 8.0) and used as a template. DNA concentrations were measured at all stages using Nano-Drap (Thermo Scientific NanoDrop 2000 Spectophotometer USA).

### Primer design

 The primer was designed on the basis of the reference sequence (*S.aureus* ATCC 14458 enterotoxin B gene, accession number AY518386.1) and analyzed by primer3 software. Multiple alignment was carried out by DNASIS MAX trial version.

### DNA amplification and sequence analysis

 For DNA amplification, the mastermix was made in 200 µl microtubes by using a 25 µl reaction mixture that contained the 1 µl DNA template, 2 U of Taq DNA polymerase, 5 µl of 10X PCR buffer II that contained 2 mM of each dNTPs, 2 mM MgCl_2_ (all reagents were from Fermantase), 10 µM of the primer pair (synthesized by Cinnagen Co., I.R. Iran) and double-distilled water, to a final volumeof 25 µl. All amplifications were carried out in a thermal cycler (Bio-Rad, C_1000_) with initial denaturation at 94°C for 5 minutes followed by 32 cycles of denaturation at 94°C for 1 minute, primer annealing at 56°C for 1 minute and extension at 68°C for 1 minute, followed by a final extension at 68°C for 7 minutes. The amplified PCR products were electrophoresed in a 1.5% agarose gel, then stained with etidium bromide (0.05 mg/ml; Sigma Aldrich). The gels were photographed under ultraviolet light using Gel Doc (Bio Rad Universal Hood II, USA). Molecular size markers (50 bp) were included in each agarose gel. The PCR product was purified and sent to the Sequencing Laboratory (Hope Generation Medical Foundation, Iran) to determine the sequence.

### Western-blotting for confirmation of Staphylococcal enterotoxin B

 In this method, SEB antibody (Abcam, Ab 15898, 500; lot 693317) was applied. The immunoblotting method was performed briefly (16) as follows: culture supernatants were concentrated by a centrifugal filter device (Amicom ultracel 10, 30, 50, and 100 kda). Protein samples were separated by SDS-PAGE and then electrophoretically transferred in a semidry transfer apparatus (Trans-Blot SD Electrophoretic Transfer Cell cat. no. 170-3940) to the PVDF membrane. The transferred proteins were bound to the surface of the membrane, which provided access for reaction with immunodetection reagents. All remaining binding sites were blocked by immersing the membrane in a solution that contained 3% bovine serum albumin (BSA) for an hour. The membrane was washed three times with phosphate buffer saline (PBS): 0.23 g NaH_2_PO_4_1.15 g Na_2_HPO_4_, 9.00 g NaCl, to which H_2_O was added to reach 1000 ml and adjusted to pH=7.2. After washing, the membrane was immersed in a primary antibody solution (1 to 15,000 diluted in BSA 0.5%) overnight. The membrane was then washed three times and the antibody-antigen complex was identified with horseradish peroxidase (HRPO) coupled to the secondary anti-IgG antibody (e.g., rabbit IgG_1_ was diluted up to 10,000 fold in 0.5% BSA). Chromogenic substrates were then used to visualize the specific bindings.

## Results

 The results of identified strains showed that 28 strains (~ 9.3%) were coagulase negative and 272 strains (~ 90.7%) were coagulase positive. The 300 *S.aureus* strains were derived from blood (63 strains), CSF (51 strains), synovial fluid (9 strains),throat swabs (63 strains), wounds (81 strains), stool (22 strains) and the genital tract (11 strains).

 The results of DNA extraction by the salting out method represented relative purity and concentration. Results of samples analyzed by the Nano-Drap method revealed an absorption ratio of 260/280 nm, which was above 1.8 and represented the desired purity of extracted DNA.

**Fig 1:**
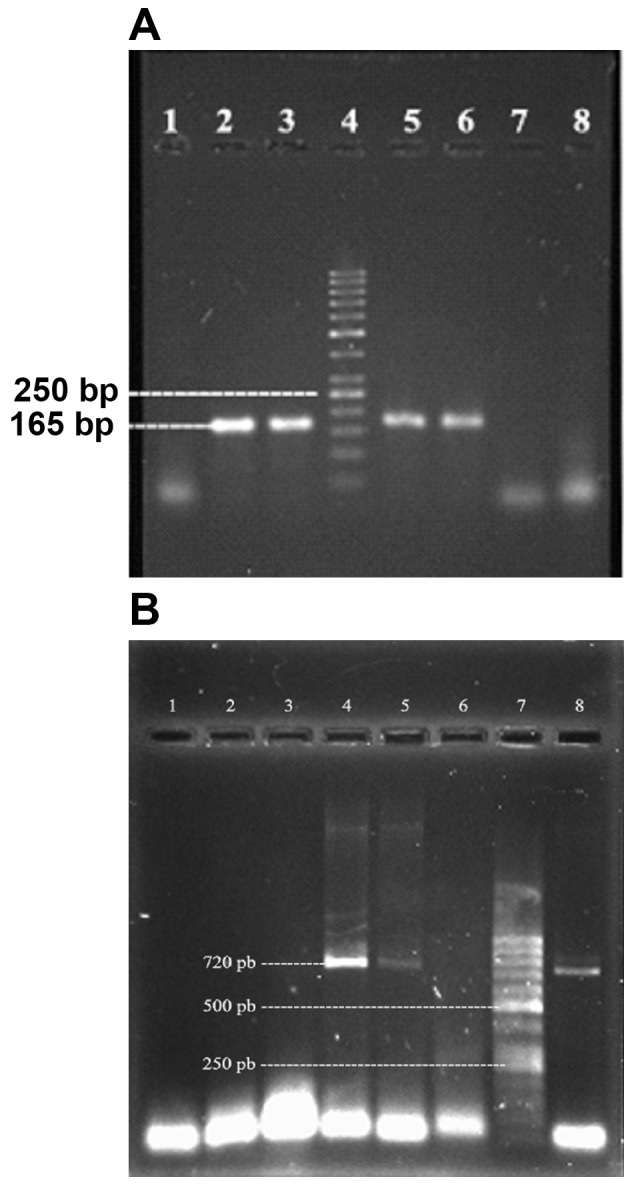
A. lanes 2, 3, 5 and 6 are 165 bp PCR product, lane 4 is a 50 bp MW standard and lanes 1, 7 and 8 are negative controls. B. lane 7 is a 50 bp MW standard, lanes 4, 5 and 8 are 720 bp and lanes 1, 2, 3 and 6 are negative controls

 Agarose gel electrophoresis of DNA extracted in this method showed favorable and sharp bands. As shown in figure 1, the results of different primers designing were as; F1-TGTATGTATGGTGGTGTAAC and R1- ATAGTGACGAGTTAGGTA; and F2-GAGAGTCAACCAGATCCTAAACCAGA and R2-TCACTTTTTCTTTGTCGTAAG which were amplified at 165bp and 720bp fragments as PCR products, respectively. In Fig 1, the result of 1.5% and 1% agarose gel electrophoresis of PCR products from primer 1 and 2 are shown in parts Aand B, respectively.

 The results indicated that the PCR method was optimized for the detection of the SEB gene. All 300 isolated strains were studied, and the primer pairs which were amplified for the 165 bp fragment as well as the primer pairs which were amplified for the 720 bp fragment were only detected in 15 out of the 300 strains of the *S.aureus* SEB Gene. The distribution of 15 *S.aureus* strains which carried the SEB gene were isolated from blood (1 strain), synovial fluid (3 strains), throat swabs (4 strains), wounds (3 strains), and stool (4 strains).

**Fig 2:**
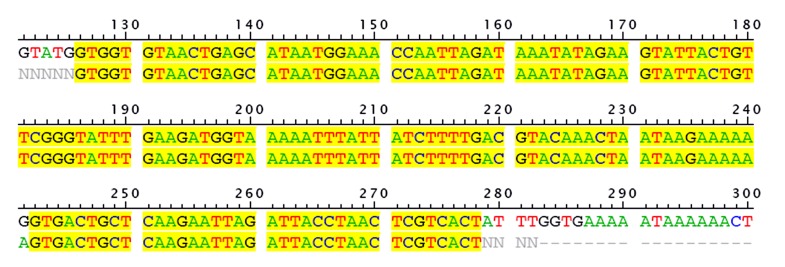
The 165 bp fragment amplified (purified PCR product) in this study was sequenced and aligned with the reference SEB gene ATCC 14458 (accession number AY518386.1). The first row sequence is related to the reference gene and the second row is the PCR product sequence derived from this research.

 These strains were maintained on culture media that contained 15% glycerol in a −80°C freezer. DNA sequencing of the amplicon 165 bp fragment SEB gene as a PCR product was carried out by the Iranian company sequencer (Hope Generation Medical Foundation, Iran). The result of the multiple alignment of the reference gene (*S.aureus* strain ATCC 14458 enterotoxin B gene, accession number AY518386.1) with sequenced PCR product is shown in Figure 2. In order to prevent contamination, each of the samples were analysed separately.

 The result of total protein measurment in the 24 hour culture supernatant of all strains that contained the SEB gene were 735 µg/ml to 7.9 mg/ ml. The result of using the centrifugal filter device (Amicom ultracel 10, 30, 50 and 100 kda) revealed that the supernatants were 10 times more concentrated than initial.

**Fig 3:**
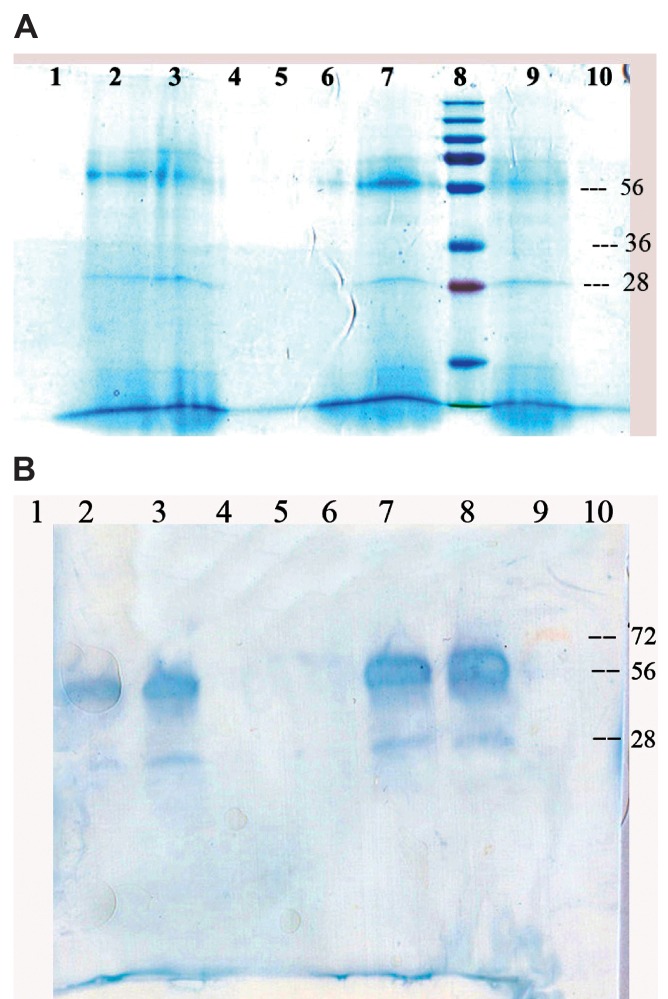
A. Lanes 2,3,7, and 9 SDS PAGE of the concentrated extracts of supernatant from four samples. B. Lanes 2,3,7 and 8 show Western-blotting of the same samples.

 SDS PAGE electophoresis of 15 µl of concentrated culture supernatant from each of the samples revealed a protein band in the area marked 28 kda and 56 kda (Fig 3A). In order to perform the Western-blotting test, an alternative SDS PAGE electrophoresis was carried out and the unstained gel (protein band) was electrophoretically transfered to PVDF (Millipore Immobilon P) paper. After that, Western-blots with specific antibody against SEB (Abcam, Ab 15898, 500; lot 693317) were carried out. The result is shown in Figure 3.

 As shown in Figure 3A, ultrafiltration (centrifugal solution over 10 kda and below 30 kda filter device) product electophoresis has made a weak 28 kda band and a strong 56 kda band. The presence of SEB in both areas was approved by Western- blot (Fig 3B).

## Discussion

*S.aureus* is a major human pathogen and several health problems caused by this pathogen have been reported worldwide. Thus, different aspects of its pathogenicity and invasiveness have been investigated by researchers. Studies have been done on antibiotic resistance (17), infection control (18), diagnostic aspects, and pathogenicity (19). However, the role of staphylococcal enterotoxins have not yet received the adequate attention of investigators and still require more research.

 In this study, both the presence of the enterotoxin B gene of *S.aureus* in clinical specimens and also its ability for toxin production was examined. The results revealed that out of 300 *S.aureus* strains isolated from clinical samples, only 15 strains (5%) contained the *ent B* gene and had the ability to produce related enterotoxin *in vitro*. This finding is based on the PCR and Western-blot methods used in this study.

 Previous studies have reported variation in the prevalence of *S.sureus* production of enterotoxin B. For example, the SEB producer among *S.aureus* isolated from basket handlers was 12%, nasal swabs of supermarket workers 2%, and clinical specimens 4% (20). This study revealed that 5% of the strains carried the *ent B* gene and were able to produce enterotoxin B. However, the pathogenesis of this toxin in organs other than the gastrointestinal tract remains unclear.

 In addition, the characteristics listed for the SEB are increasing importance. For example, SEB is a powerful superantigen and may have synergistic effects with gram-negative bacterial endotoxins(21). In addition, SEB is a potent mitogen that elicits life threatening and T- cell proliferation, and cytokine production (22). Hence, a diagnostic microbiology laboratory should be equipped to detect enterotoxigenicity in addition to bacterial identification. In this way, new methods can be designed to treat infections better, prevent undue use of antibiotics, and prevent the emergence of antibiotic resistance. *S.aureus* is a major pathogen responsible for both nosocomial and community acquired infections ranging from localized skin infections to severe systemic diseases such as septicemia (23). 

 Recent genetic advances have established several methods which have enabled identification of many pathogenic factors and characterization of clinical isolates (24). However, detection and amplification reaction tests are still improving and fu- ture developments coupled with other techniques might provide more broadly usable tools. 

 Symptoms of toxic shock syndrome (TSS) were documented for the first time by the famous Greek physician Hippocrates (c. 460-377 B.C.). From that time onward, *S.aureus* has remained a major health problem. This indicates two important facts: one is the diversity of pathogenic factors related to *S.aureus* and the other the lack of properly designed methods for diagnosis and effective protection. Thus, there is need for extensive studies on the staphylococcal toxin and other pathogenic factors related to it.

 In this study, we found multiple alignments of the amplified fragment (165 bp) with reference *S.aureus* strain ATCC 14458 enterotoxin B gene (GeneBank: AY518386.1) which were highly matached (max score 98%). It began at the 126 nucleotide position in the reference *S.aureus* strain ATCC 14458 enterotoxin B gene. As shown in Fig 2, there is only one nucleotide difference in position 141, code AGT for amino acid serine instead of code GGT for amino acid glycine. Blast homology analysis of the 165 bp fragment showed 98% similarity to all SEB sequences in the gene bank data base. Despite the fact that the enterotoxin B gene in native strains has a one amino acid difference with the reference strain, it reacted well with the specific antibody (Abcam, Ab 15898, 500; lot 693317).

## Conclusion

 The results in this study conclude that the presence of the SEB gene among clinical isolates is 5%, which is noticeable. In addition, all strains containing SEB gene have produced detectable amounts of enterotoxin B in cultured media. Thus,it is important to note that in the clinical cases with transient staphylococcal infection, patients may have a small amount of enterotoxin B secreted in their body. This can lead to serious consequences such as the enhancement of the lethality of the endotoxin, which can promote fatal diseases such as TSS. These complications caused by enterotoxin B can be prevented by comprehensive and sensitive detection techniques combined with the conventional identification of *S.aureus* used routinely in microbiology laboratories.
